# The History of Antibiotic Treatment of Osteomyelitis

**DOI:** 10.1093/ofid/ofz181

**Published:** 2019-04-08

**Authors:** Nicolás W Cortés-Penfield, Prathit A Kulkarni

**Affiliations:** 1 Infectious Diseases Section, Department of Medicine, Baylor College of Medicine, Houston, Texas; 2 Medical Care Line, Michael E. DeBakey Veterans Affairs Medical Center, Houston, Texas

**Keywords:** antibiotic, antimicrobial, bone and joint infection, bone infection, history, oral antibiotics, osteomyelitis

## Abstract

Antibiotic treatment of osteomyelitis has evolved substantially over the past 80 years. Traditional teachings (eg, that antimicrobials must be given parenterally, selected based upon ratios of achieved bone vs serum drug levels, and continued for 4–6 weeks) are supported by limited data. New studies are challenging this dogma, however. In this review, we seek to contextualize the discussion by providing a narrative, chronologic review of osteomyelitis treatment spanning the pre-antibiotic era through the present day and by describing the quality of evidence supporting each component of traditional osteomyelitis therapy.

## Teaser

Recent studies challenge traditional dictums about the appropriate choice, route, and duration of antibiotics for osteomyelitis. In this paper, we provide a narrative review of the history of osteomyelitis treatment to critically examine the quality of evidence behind these traditional approaches to osteomyelitis therapy

Despite being a common reason for infectious disease consultation, osteomyelitis remains relatively understudied. For example, at the time of this writing, a PubMed search yields 61 randomized controlled trials published between 2000 and 2018 containing the keyword “osteomyelitis.” By comparison, a similar search for acute gastroenteritis yields 84 results, cellulitis 133 results, the common cold 218 results, and urinary tract infection 597 results.

The results of this neglect are 2-fold. First, the full scope of the human cost of osteomyelitis remains unclear. Epidemiologic studies of osteomyelitis are rare and mostly restricted to subtypes of osteomyelitis (eg, osteomyelitis in a specific population, at a specific anatomic site, or due to a specific organism) [1–3]. The broad population-based data that do exist suggest that osteomyelitis is common and increasing in incidence, in part due to a sharp uptrend in osteomyelitis associated with diabetes mellitus [4].

Second, clinicians lack a standardized, well-established, and evidence-based approach to antibiotic treatment of osteomyelitis. Traditional teaching generally states a version of the following: After surgical debridement and removal of infected foreign material, medical management of osteomyelitis should constitute at least 4–6 weeks of parenteral antibiotic therapy with an agent that concentrates at high levels in bone compared with levels achieved in serum (also termed “bone penetration”) [5, 6]. The data to support each tenet of this teaching are limited, however, and new data are emerging that challenge existing beliefs about osteomyelitis treatment [7, 8]. The purpose of this manuscript is to describe the historical development of osteomyelitis treatment and describe the evidentiary basis for each component of the traditional treatment approach.

## METHODS

We reviewed the medical literature to construct a narrative overview of the antibiotic treatment of osteomyelitis. To identify references, we searched the PubMed database using combinations of keywords, including “osteomyelitis,” “bone infection,” “antibiotic,” “antimicrobial,” “oral,” and “duration” for English-language articles readily available through the Texas Medical Center online library. We identified comparative and noncomparative studies of antibiotic treatment of osteomyelitis, as well as a representative sample of reviews of osteomyelitis published between 1919 and 2018, and used article citations to identify additional references. In some instances, we present descriptive statistics and secondary analyses of data reported in these manuscripts. These analyses used the chi-square test or Fisher exact test, as appropriate, to compare categorical data and were conducted in SAS 9.3 (Cary, NC).

## RESULTS

### Prehistory–1940: Osteomyelitis Treatment in the Pre-antibiotic Era

An Egyptian surgical treatise authored between 2500–3000 BCE provides the first written description of osteomyelitis in humans. This text describes a man with neck stiffness following a penetrating trauma with exposure and perforation of 1 of the cervical vertebrae. The author renders a doubtful prognosis, advising that in such cases the wound be bound with fresh meat and that the patient “[be moored] at his mooring stakes until the period of his injury passes by” (ie, the clinician can only wait and see what happens) [9].

Between 3000 BCE and the early 20th century, osteomyelitis was a surgical disease characterized by great morbidity and mortality. In 1919, the editor of *Annals of Surgery* writes, “[Osteomyelitis] is tenacious in its course, and its final cure is always difficult, sometimes impossible to secure” [10]. He later summarizes the state of osteomyelitis treatment as follows:

When, however, chronic osteomyelitis has developed, what is to be done? It cannot be too strongly emphasized that osteomyelitis is controllable only by an operative interference which involves the free exposure of the osteitic focus throughout its whole extent, in the complete removal of the lesions; finally, in the securing of the cicatrization after the bone cavity has been rendered shallow and fully open. The sooner this is done, the better; that is to say, the procedure should be resorted to as soon as the diagnosis is established. [10]

Adjunct therapies employed during this period included irrigation of surgical wounds with Dakin’s solution and maggot therapy, practices that never underwent rigorous study compared with surgical management alone [11, 12]. Interestingly, in 1934 Buchman describes instillation of bacteriophage cocktails into the surgical space as another adjunctive therapy for osteomyelitis [12]. He writes that a number of strains of phage active against staphylococci had been identified, and that while the technique was not consistently helpful, it seemed more effective than irrigation of the wound with Dakin’s solution, more comfortable for the patient, and associated with shorter periods of convalescence. This approach appears to have been abandoned in the West after the advent of antibiotics, as no subsequent clinical data regarding phage therapy appeared in the English-language literature over the next several decades. A recent case report of successful phage therapy for osteomyelitis associated with a diabetic foot infection suggests renewed interest in phages for bone and joint infections in the modern era of antimicrobial resistance [13].

The early 20th century was also the time when the clinical differentiation between acute and chronic osteomyelitis first appeared. Writing in 1922, Wilensky defines acute osteomyelitis as arising from bacteremia leading to thromboembolism and septic thromboarteritis or thrombophlebitis of the intraosseus vasculature, which then go on to produce bone necrosis [14]. This definition was based upon careful observation of plain radiographs showing anatomic patterns of disease reflecting interruption at various “fixation points” in the bony vasculature (ie, septic thromboses). These studies generated our current understanding of osteomyelitis pathophysiology, in which acute osteomyelitis is thought to have a bacteremic origin, whereas chronic osteomyelitis results from contiguous spread of infection from a wound or another established infectious focus. Along with this distinction in pathophysiology, Wilensky describes a distinction in prognosis, writing that while a diagnosis of acute osteomyelitis leaves some hope for clinical cure, “[it] is beyond dispute that in some cases of osteomyelitis a definite end result cannot be said ever to exist…. These are those chronic cases of osteomyelitis with persistent, uncontrollable discharging fistulae which seem to defy our best efforts at treatment” [14].

### 1940–1950s: Initial Experiences With Sulfonamides, Streptomycin, and Penicillin

The earliest report of systemic antimicrobial treatment of osteomyelitis that we could locate was Penberthy and Weller’s 1941 case series of 19 children with acute osteomyelitis treated with sulfapyridine and sulfathiazole [15]. These patients had infections primarily due to *Staphylococcus aureus* and received sulfonamides orally for a median (range) of 11 (3–37) days. Sixteen of 19 achieved clinical cure or marked improvement. Although 3 patients had poor outcomes, none died; the authors note this with surprise, citing a mean 13.5% mortality rate among contemporary case series of osteomyelitis treated without antibiotics. In 1947, Lamphier et al. report using intramuscular streptomycin for 5–7 days in 4 cases of chronic osteomyelitis due to gram-negative bacteria in young men who had sustained bullet and shrapnel wounds while fighting in World War II [16]. Again, the authors observed marked clinical improvement in each case.

The true revolution occurred with the introduction of penicillin. Between 1945 and 1946, 4 groups published case series comprising a total of 135 patients given intramuscular penicillin for treatment of osteomyelitis [17–20]. Most patients were under 15 years of age, and 39%–75% had infections due to *S. aureus*. Courses of treatment ranged from less than 1 week to more than 1 month. In the study by Higgins et al., the median duration of therapy among 31 patients was 14 days. There were no deaths in any of the case series; clinical cure rates ranged from 82% to 100%. The authors comment in these case series on how quickly systemic symptoms subsided and local and metastatic progression of infection ceased after patients received penicillin and surgical source control. Penicillin had transformed the prognosis of osteomyelitis.

It is interesting to note that, in these earliest experiences with beta-lactam antibiotics for osteomyelitis, short-course therapy yielded excellent clinical outcomes. Moreover, 62 (45%) of the patients in these case series had chronic osteomyelitis, and their outcomes were similar to those with acute infections. An important limitation of these data (and indeed, many of the published studies of osteomyelitis) is that the durations of follow-up were often either not reported or were only a few months. Therefore, they may overestimate the clinical success rates achieved by having failed to capture late relapses that occurred in the 1–2 years after treatment. Despite this caveat, these data do challenge the notion that acute and chronic osteomyelitis are fundamentally and incomparably different disease states.

### 1960–1970s: Oral Tetracyclines and Beta-Lactams and the Codification of Current Dogma

Penicillin-resistant *S. aureus* was widespread by the 1960s, turning interest to newer agents [21]. In 1962, Cullen and Hargadon published the first comparative study to support use of tetracyclines for treatment of osteomyelitis, in a retrospective cohort of 55 patients given either oral tetracycline or intramuscular penicillin for 3–4 weeks. They found lower rates of infectious complications such as abscess or sequestra formation in the patients given tetracycline compared with penicillin (15% vs 54%, *P* = .0049) and higher rates of hospital discharge within 30 days (60% vs 26%, *P* = .02) [22]. An important caveat of this study was that penicillin-resistant *S.* aureus accounted for more than half of cases with a microbiologic diagnosis, suggesting that bacterial nonsusceptibility may have confounded the high rates of treatment failure in the penicillin arm. Interestingly, patients with penicillin-sensitive *S. aureus* given penicillin had similarly high rates of complications, a finding not observed in other studies from this era and for which a clear reason was not noted. In a subsequent small randomized clinical trial, oral tetracycline yielded markedly lower rates of clinical improvement than cloxacillin in chronic osteomyelitis due to *S. aureus* sensitive to both agents (2/21 vs 14/27 cases, *P* = .002). Inquiry into tetracycline use for osteomyelitis treatment was seemingly abandoned after these 2 publications [23].

Simultaneously, other clinicians began publishing their experiences with penicillinase-resistant penicillins and cephalosporins. Six additional studies, including 2 clinical trials and 4 case series encompassing a total of 152 patients, appeared in the literature during this period [24–29]. These studies primarily enrolled children (80% of participants); acute osteomyelitis predominated (76% of cases), and *S. aureus* remained the pathogen most often isolated. Routes of antibiotic administration were intramuscular in 1 study, intramuscular or intravenous until systemic signs of infection improved, followed by stepdown to oral therapy in 3 studies, and all-oral therapy in 2 studies. Cloxacillin and cephalexin were the most commonly used oral drugs. Durations of therapy ranged from 3 to 8 weeks in the studies of acute osteomyelitis and 3 to 18 months for cases of chronic osteomyelitis. It is unclear to us from reviewing this literature why treatment durations for chronic osteomyelitis dramatically increased during this period. One possibility is that clinicians opted for longer treatment durations because of prior knowledge about poor outcomes in chronic osteomyelitis in the pre-antibiotic era. These studies universally reported good treatment outcomes, with initial significant clinical improvement or cure in a mean 98% of patients and a mean subsequent relapse rate of 7%, which did not differ between acute and chronic cases. Periods of follow-up varied widely, but most fell between 6 months and 2 years.

Our modern approach to osteomyelitis therapy appears to have originated during this time period. This codification is evident in a review on the topic published by Waldvogel et al. in the *New England Journal of Medicine* in 1970 [30]. Interestingly, the authors deemphasize the importance of stratifying osteomyelitis by acuity, writing that “there is no abrupt shift from acute to chronic disease but rather a gradual blending of one into the other. In our experience, the pathological findings often prove ambiguous…. Distinction between acute and chronic osteomyelitis on clinical grounds [is] frequently difficult as well.” The basis for the shift from the prior view of the pathogenesis of acute vs chronic osteomyelitis as clearly separate to this view is uncertain, and the conceptualization of osteomyelitis acuity as a gradient without clear demarcations does not appear to have persisted in the literature after this review. In fact, there is a notable lack of studies directly comparing patients with acute vs chronic osteomyelitis, or children vs adults with osteomyelitis of either form. Whether the recalcitrant nature of chronic contiguous osteomyelitis in adults vs the acute hematogenous form in children is due to fundamental differences in the pathophysiology of these infections, rendering their studies totally nongeneralizable, or whether the prognosis seems poorer in adults simply because adults more often have comorbidities predisposing to recurrent infections (eg, neuropathy and vascular insufficiency) remains an open question. In any case, Waldvogel and colleagues define “adequate treatment” of osteomyelitis as receipt of a high-dose parenteral beta-lactam for at least 4 weeks and present their clinical experiences with 62 cases of hematogenously acquired osteomyelitis, showing better clinical results among patients who received their definition of adequate treatment. Notably, the authors did not cite the earlier studies reporting good outcomes with oral antibiotics and shorter courses of therapy.

Waldvogel et al. also mention studies of antibiotic levels of bone in their antibiotic selection criteria for “adequate treatment,” thereby introducing the concept of antibiotic bone penetration into mainstream discourse. These studies typically involved assaying antibiotic levels in homogenized samples of bone from otherwise healthy adult volunteers administered antibiotics before elective hip arthroplasties, and thereafter establishing the ratios of levels of drug achieved in bone vs levels simultaneously achieved in serum [31]. Limitations of bone penetration studies include uneven distribution of antibiotics between compartments within bone, uncertainty as to which sites pathogens inhabit within diseased bone (eg, within osteoblasts vs bone matrix), and uncertainty as to the clinical significance of these variables. Although the importance of bone penetration in selecting optimal antibiotics for osteomyelitis is frequently discussed, we were unable to identify any comparative clinical data to support this concept nearly half a century later. This is perhaps unsurprising given that a systematic review found that most antibiotic classes achieve similar bone-to-serum ratios, with the exception of oral penicillins and cephalosporins, whose clinical track records for treating osteomyelitis are nevertheless comparable [31].

### 1980–1990s: The Fluoroquinolone Era

In 1980, the advent of broad-spectrum fluoroquinolones revolutionized anti-infective therapy. Writing in 1989, Norby reports the results of 9 studies (total n = 182) of oral ciprofloxacin for treatment of chronic osteomyelitis due to *Pseudomonas aeruginosa*, other gram-negative organisms, and *S. aureus* [32]. In these studies, oral ciprofloxacin, most frequently given in durations between 4 weeks and 6 months, cured osteomyelitis in 70% of cases and led to significant improvement in another 5% over periods of follow-up ranging from several weeks to over 1 year.

Concurrently, 3 randomized control trials with a total of 128 patients compared the efficacy of 4–6 weeks of an oral fluoroquinolone with a similar duration of parenteral antibiotics (typically a third-generation cephalosporin with or without an aminoglycoside) [33–35]. Both within each study and when aggregated, oral fluoroquinolones produced similar rates of cure (cumulative rates of 85% vs 86%, *P* = .96) and relapse (cumulative rates of 22% vs 15%, *P* = .34), with infections due to *P. aeruginosa* and *S. aureus* accounting for the majority of clinical failures and relapses in the fluoroquinolone arms.

Rabbit experiments conducted in the 1980s showed greater rates of osteomyelitis cure with vancomycin given in combination with rifampin vs vancomycin alone, leading Dr. Carl Norden and colleagues to perform a clinical trial comparing nafcillin monotherapy with nafcillin plus rifampin in patients with chronic *S. aureus* osteomyelitis [36, 37]. Of note, none of these patients had prosthetic device infections. Norden et al. observed clinical cure in 8/10 patients given the combination treatment vs 4/8 patients given nafcillin monotherapy. Van der Auwera and colleagues found similar results in a clinical trial comparing oxacillin or vancomycin plus either rifampin (study arm) or placebo (placebo arm) in patients with infections due to *S. aureus*; among the 23 included cases of osteomyelitis, 5/6 clinical failures occurred in the placebo arm [38]. These studies were followed by much literature describing the value of adding rifampin for bone and joint infections, and subsequent retrospective studies and randomized controlled trials showed excellent treatment outcomes with oral rifampin combinations vs traditional parenteral therapies for osteomyelitis, including rifampin-trimethoprim-sulfamethoxazole, rifampin-linezolid, rifampin-clindamycin, and rifampin combined with a fluoroquinolone [39–43].

A small trial randomizing patients with orthopedic device–associated staphylococcal infections to either ciprofloxacin monotherapy or ciprofloxacin plus rifampin found greater cure rates with combination therapy (100% vs 58%, *P* = .02). A subsequent retrospective study of orthopedic device–associated staphylococcal infections found that rifampin use was associated with fewer relapsed infections [44, 45]. The data to support the value of rifampin in osteomyelitis without infected hardware are less robust. A retrospective study of 35 patients with *S. aureus* vertebral osteomyelitis observed more frequent administration of rifampin to patients who had cured vs relapsed infections (50% vs 0%, *P* = .06) [46]. On the other hand, in a retrospective review of oral stepdown regimens for osteomyelitis, patients switched to oral regimens without rifampin achieved rates of cure similar to those achieved by patients given rifampin combinations (7/8 vs 10/11 cured) [47]. Although the addition of rifampin to a fluoroquinolone for treatment of osteomyelitis due to *S. aureus* appears to be important given the poor outcomes associated with fluoroquinolone monotherapy for that particular organism, the general value of adding rifampin to another agent for osteomyelitis absent a prosthetic device infection has not yet been firmly established [48]. INTREPID, a large multicenter randomized controlled trial examining adjunctive rifampin vs standard antibiotic therapy for diabetic foot osteomyelitis (NCT03012529), is underway and should clarify the role of rifampin in osteomyelitis not involving infected prosthetic material.

### 2000–Present: Further Evidence for Oral Antibiotic Therapy and Answering the Question of Treatment Duration

Evidence to support to oral treatment of osteomyelitis has increasingly mounted over the past 2 decades. Pediatric infectious disease experts spearheaded this shift in practice after recognizing that central venous catheters for parenteral antibiotic therapy in osteomyelitis are associated with complication rates of up to 40%, with complications including catheter malfunction or displacement, catheter site infections, and catheter-related bloodstream infections [49].

Multiple small observational studies and a clinical trial (range, 20–95) indicated that oral agents with high bioavailability such as trimethoprim-sulfamethoxazole, clindamycin, amoxicillin-clavulanate, and first-generation cephalosporins yield high cure rates in acute osteomyelitis [50–54]. Two large retrospective cohort studies that examined treatment of >4000 children found that fully parenteral antibiotic therapy for acute osteomyelitis was not associated with lower rates of treatment failure when compared with inpatient parenteral treatment followed by oral antimicrobial therapy after discharge [55, 56]. The study by Keren et al. also found that patients given outpatient parenteral therapy were more likely to have emergency room visits and readmissions, primarily because of catheter-related complications. The authors concluded that prolonged parenteral therapy for osteomyelitis in children should be avoided when effective oral options are available.

In addition to the rifampin combination studies discussed in the prior section, more recent work on oral antibiotic therapy for adults with osteomyelitis has included several observational studies and a randomized controlled trial (mean n = 40; range, 9–77) supporting oral linezolid’s equivalency to parenteral therapy [57–61]. Another observational study supported the use of oral metronidazole, fluoroquinolones, trimethoprim-sulfamethoxazole, clindamycin, and amoxicillin-clavulanate for adult patients with osteomyelitis, reporting a cumulative treatment success rate over 80% [62]. These studies, and the pharmacokinetic parameters of parenteral and oral antibiotics often used for osteomyelitis, are comprehensively summarized in the seminal 2012 review by Spellberg and Lipsky [63]. In 2013, a Cochrane systematic review summarized data from 4 clinical trials of osteomyelitis in adults (total n = 150) and did not find any difference between oral and parenteral therapy with regards to clinical response 1 year after the end of treatment [64].

Finally, in 2019, the Oral Versus Intravenous Antibiotics for Bone and Joint Infections (OVIVA) randomized controlled trial reported the outcomes of 1054 patients with osteomyelitis given either oral or parenteral antibiotic therapy [8]. The authors found no difference in rates of definite treatment failure within 1 year of randomization, even in a “worst-case scenario” analysis in which all patients with incomplete follow-up were assumed to have had treatment failure on oral therapy and success with parenteral therapy. Patients received a broad range of oral antimicrobials including oral beta-lactams and tetracyclines and had no heterogeneity in outcome based on either the infecting pathogen or the recommended oral antimicrobial. OVIVA’s strengths included its pragmatic design, enrollment of a large proportion of patients with prosthetic device infections, and few exclusion criteria (most notably, *S. aureus* bacteremia or another infection specifically requiring parenteral therapy).

The other approach researchers have taken to address the burdens of prolonged parenteral antibiotic therapy is to reevaluate the necessary duration of antibiotics for osteomyelitis. Some experts have suggested that osteomyelitis limited to cortical bone (ie, Cierney-Mader stage II or “superficial” osteomyelitis) may be adequately treated with 2 weeks of antibiotic therapy [65, 66]. However, we have not been able to locate any published clinical data to support this assertion. A 2010 randomized controlled trial found that 131 children with acute osteomyelitis did equally well with 20 vs 30 days of antimicrobial therapy [67]. In adults, 2 randomized controlled trials (n = 40 and n = 359, respectively) showed the noninferiority of 6 vs 12 weeks of antimicrobial therapy for diabetic foot and vertebral osteomyelitis, respectively [68, 69]. However, in the study of vertebral osteomyelitis, receipt of 6 vs 12 weeks of antibiotics did not meet noninferiority criteria in all subgroups (eg, patients with diabetes or immunosuppression). In addition, a subsequent retrospective study of 314 patients with microbiologically confirmed vertebral osteomyelitis found that extended durations (≥8 weeks) of antibiotic therapy were associated with lower rates of recurrent infection among patients with risk factors for recurrence, which included infection with methicillin-resistant *S. aureus* (MRSA), undrained paravertebral abscess, and end-stage renal disease [70]. A 2019 meta-analysis reviewed 5 randomized controlled trials and 10 observational studies on antibiotic duration in osteomyelitis, concluding that the safety of shorter-course therapy is clearer for children with acute osteomyelitis, whereas data are less certain for adults and may point toward a potential benefit of longer durations of treatment for adults with vertebral osteomyelitis and infections due to MRSA [7].

## CONCLUSIONS

Although antibiotic treatment of osteomyelitis has significantly advanced over the last 80 years, standard approaches to treatment of the condition do not appear to have an extensive evidentiary basis. We find few historical data to support dictums such as the necessity of parenteral therapy, the universal necessity of giving at least 4–6 weeks of antibiotics, or the criticality of selecting antibiotics with superior “bone penetration.”

What approaches, then, does the balance of the osteomyelitis literature support? First, in the case of oral vs parenteral therapy for osteomyelitis, the highest-quality available data demonstrate the noninferiority of oral therapy. In our view, the literature does not support a general preference for parenteral antibiotics in osteomyelitis. Second, few human clinical outcomes data support the relevance of antibiotic “bone penetration”; additionally, studies of drug levels in bone yielded similar results across most antibiotic classes, so the value of this frequently taught clinical pearl seems limited. Finally, no high-quality data compare the standard 4–6 weeks of therapy with shorter regimens for adult osteomyelitis. Early historical data suggest that there is likely a subgroup of adults with osteomyelitis at low risk of recalcitrant disease who would do well with less than 4 weeks of therapy. However, the work of Park et al. suggests that 4–6 weeks may be inadequate for patients at high risk of recurrent infections, such as those with infection due to MRSA, end-stage renal disease, and uncontrolled infectious foci [70]. Although it seems prudent to continue prescribing 4 or more weeks of antibiotics for adults with osteomyelitis until more data are available, large comparative studies addressing the adequacy of shorter durations of therapy, particularly in cohorts of patients stratified by risk of treatment failure, are clearly needed.
